# Association Between the Individual and Combined Effects of the *GSTM1* and *GSTT1* Polymorphisms and Risk of Leukemia: A Meta-Analysis

**DOI:** 10.3389/fgene.2022.898937

**Published:** 2022-07-22

**Authors:** Ting Hu, Guozhong Zhou, Wenjin Li

**Affiliations:** ^1^ Department of Hematology, Pingxiang People’s Hospital, Pingxiang, China; ^2^ Department of Cardiology, Pingxiang People’s Hospital, Pingxiang, China

**Keywords:** *GSTM1*, *GSTT1*, polymorphism, FPRP, BFDP, leukemia

## Abstract

**Background:** Fourteen meta-analyses reported the individual effects of the *GSTM1* and *GSTT1* polymorphisms on leukemia risk. However, over 40 studies were not included in previously published meta-analyses. Moreover, one key aspect was that previous meta-analyses did not conduct the false-positive test on the aforementioned issues. Furthermore, previous meta-analyses did not observe the combined effects of *GSTM1* present/null and *GSTT1* present/null polymorphism with leukemia risk. Therefore, we conducted the current study to further analyze these associations.

**Objectives:** This study aimed to investigate the association between the individual and combined effects of the *GSTM1* present/null and *GSTT1* present/null polymorphisms and the risk of leukemia.

**Methods:** A meta-analysis was performed applying Meta-analyses of Observational Studies in Epidemiology (MOOSE) guidelines. Moreover, false-positive report probability (FPRP) and Bayesian false discovery probability (BFDP) were applied to investigate the false-positive results.

**Results:** The individual *GSTM1* and *GSTT1* null genotypes and combined effects of the two genes were associated with a significantly increased leukemia risk in overall and several subgroup analyses, such as Asians, Caucasians, and so on. Then, further analysis was conducted using FPRP and BFDP. Significant associations were considered as “positive” results on the *GSTM1* null genotype with leukemia risk in overall populations (FPRP < 0.001 and BFDP = 0.006), Asians (FPRP < 0.001 and BFDP < 0.001), and East Asian population (FPRP < 0.001 and BFDP = 0.002). For the *GSTT1* null genotype, significant associations were regarded “positive” results in overall populations, acute myeloid leukemia (AML), Asians, and East Asian population. For the combined effects of the *GSTM1* and *GSTT1* polymorphisms, significant associations were also considered “positive” results in the overall analysis of Asians, Indians, and East Asian population.

**Conclusion:** This study strongly indicates that the individual *GSTM1* and *GSTT1* null genotypes and combined effects of the two genes are associated with increased leukemia risk in Asians, especially in the East Asian population; the *GSTT1* null genotype is associated with increased AML risk; the combined effects of the two genes are associated with increased leukemia risk in Indians.

## Introduction

Leukemia, commonly diagnosed in childhood, is a complex and heterogeneous disease caused by irreversible genetic lesions in initially normal hematopoietic cells (Bloomfield et al., 2001). Chronic myeloid leukemia (CML) is a clonal, myeloproliferative disease characterized by the accumulation of myeloid precursors in the bone marrow, blood, and body tissues. It is a relatively rare disease worldwide, accounting for approximately 14% of all types of leukemia (Quintás-Cardama and Cortes, 2006). The highest incidence rate is found in males of all age groups, and the fact remains to be explained (Henderson et al., 1990; Goyette et al., 1994; Pui, 2000; Pui et al., 2000; Pui et al., 2002; Pui et al., 2006; Hirschhorn et al., 2002).

The glutathione S-transferases (*GSTs*) are a family of multifunctional enzymes, which play an important role in the detoxification of toxic, potentially carcinogenic compounds and a series of basic physiological processes of the human body (Benjamini and Hochberg, 1995; Hayes et al., 2005; Udomsinprasert et al., 2005). The *GST* family is divided into seven categories of genes in humans according to their primary structure (Curran et al., 2000).

The *GSTM1* and *GSTT1* polymorphisms have been identified, resulting in possibly impaired activity for the elimination of carcinogenic compounds and increased risk of cancer (Hayes and Strange, 2000). The *GSTM1* and *GSTT1* genes are located on chromosome 1 (1p13.3) and chromosome 22 (22q11.2), respectively (Hayes and Strange, 2000). Polymorphisms in both *GSTM1* and *GSTT1* result in gene deletions (null genotype), resulting in loss of expression and enzyme activity loss (Seidegård et al., 1988; Hayes and Strange, 2000). The lack of enzymatic activity may lead to the occurrence of cancer.

Fourteen meta-analyses (Ye and Song, 2005; Das et al., 2009; Zintzaras, 2009; Vijayakrishnan and Houlston, 2010; Tang et al., 2013; He et al., 2014a; He et al., 2014b; Ma et al., 2014; Moulik et al., 2014; Tang et al., 2014; Xu and Cao, 2014; Li et al., 2018; Zhao et al., 2018; Wang et al., 2019) reported the individual effects of the *GSTM1* and *GSTT1* polymorphisms with leukemia risk. However, over forty studies were not included in previously published meta-analyses. Moreover, one key question was that previous meta-analyses did not conduct the false-positive test on the above issues. Furthermore, previous meta-analyses did not perform the combined effects of *GSTM1* present/null and *GSTT1* present/null polymorphisms with leukemia risk. Therefore, we conducted the current study to further analyze these associations.

## Materials and Methods

### Identification and Eligibility of Relevant Studies

A comprehensive literature search was conducted applying the PubMed, EMBASE, ISI, CNKI, and WanFang databases for relevant articles published (the last search update was 26 February 2022). The search strategy (it was designed to be sensitive and broad) was as follows (glutathione S-transferase T1 OR *GSTT1* OR glutathione S-transferase M1 OR GSTM1) AND (polymorphism OR genotype OR allele OR variant OR mutation) AND (leukemia OR leukaemia). In addition, studies were also identified by a search of the reference lists of reviews and retrieved studies. Moreover, all eligible studies were retrieved, and their bibliographies were checked for other relevant publications.

### Inclusion Criteria

Inclusion criteria were as listed below: 1) Case–control or cohort studies; 2) publications on the individual or combined effects of *GSTM1* present/null and *GSTT1* present/null polymorphisms with leukemia risk; and 3) complete genotype data between leukemia cases and controls. Exclusion criteria were as listed below: 1) Duplicate genotype data; 2) no case–control studies; 3) Meta-analyses, reviews, or letters; and 4) other SNPs.

### Data Extraction and Quality Score Assessment

Data were extracted independently by two investigators according to the inclusion criteria. Supplementary Table S1 lists the information on data extraction. Ethnicity was categorized as “Caucasian,” “Asian,” “Indian,” “African,” and mixed populations. “Indian” mainly came from India and Pakistan. The ethnicity was considered as “mixed population” when one study did not state which ethnic groups were included or if it was impossible to separate participants based on phenotype.

The scale of quality assessment criteria was designed based on one previous meta-analysis (Thakkinstian et al., 2011) (Supplementary Table S2). Studies scoring > 9 were considered high quality.

### Statistical Analysis

Crude odds ratios (ORs) with 95% confidence intervals (CIs) were applied to evaluate the associations between the individual and combined effects of *GSTM1* and *GSTT1* polymorphisms on leukemia risk. Between-study heterogeneity was assessed by applying the *Q* statistic and *I* ^2^ value. A random-effect model (DerSimonian–Laird model) (DerSimonian and Laird, 1986) was applied if *p* < 0.10 and/or *I* ^2^ > 50%; otherwise, a fixed-effect model (Mantel–Haenszel method) was used (Mantel and Haenszel, 1959). Subgroups were conducted by ethnicity, geographic region, and type of leukemia. In addition, a meta-regression analysis was performed to explore the source of heterogeneity. Sensitivity analysis was performed by removing a single study each time and excluding low-quality studies. Publication bias was calculated using Begg’s funnel plot (Begg and Mazumdar, 1994) and Egger’s regression asymmetry test (Egger et al., 1997). If publication bias existed, a nonparametric “trim and fill” method (Dual and Tweedie, 2000) was applied to add missing studies. Moreover, we used the following criteria to investigate the false significant results: false-positive report probability (FPRP) < 0.2 and Bayesian false discovery probability (BFDP) < 0.8 because FPRP and BFDP values can clarify the probability of no true association between genetic association and disease risk. All statistical analyses were calculated using STATA version 12.0 (STATA Corporation, College Station, TX, United States).

## Results

### Study Characteristics

**FIGURE 1:**
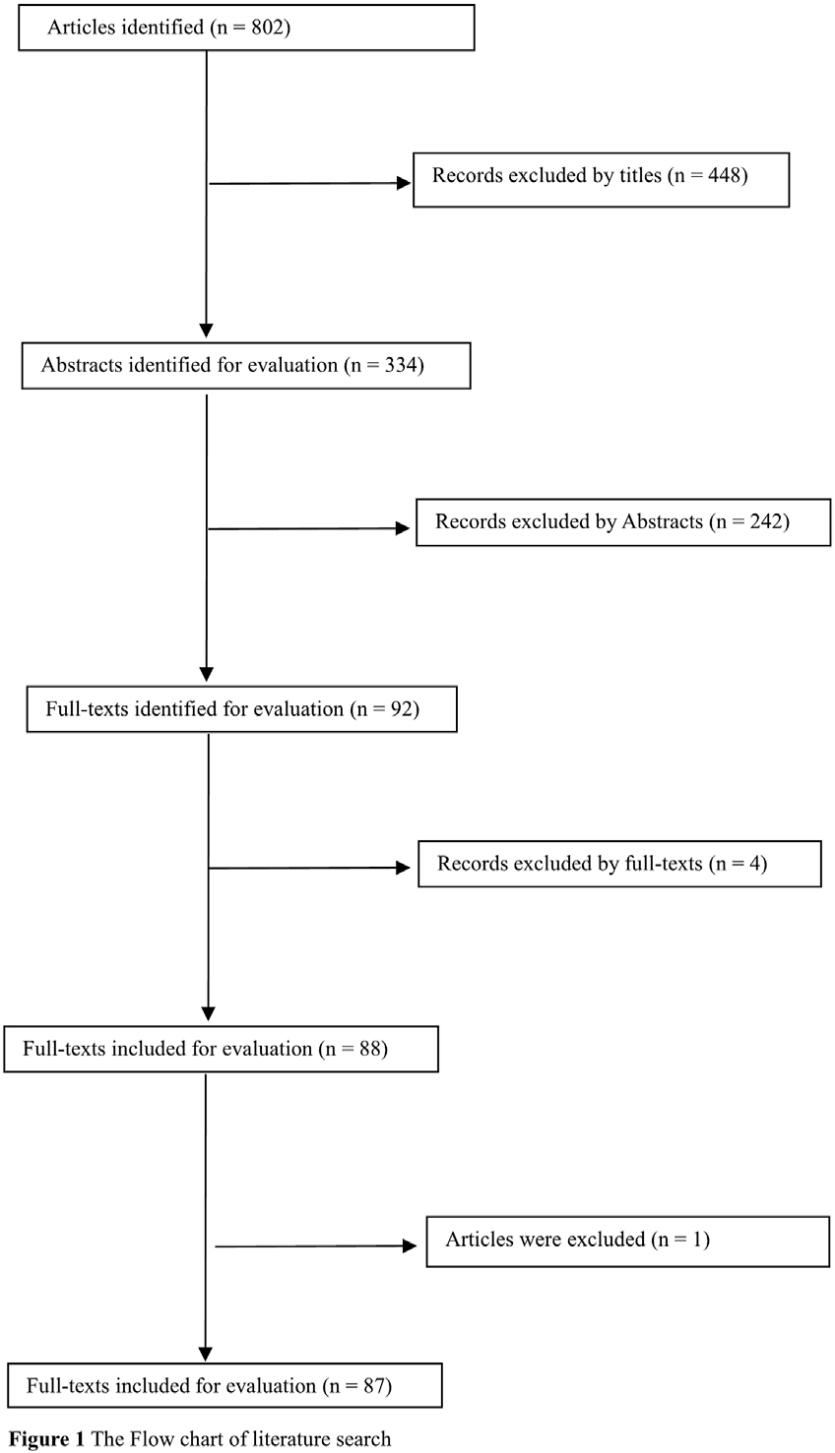
The flow chart of literature search.

Overall, 802 articles were identified. Of these, 694 were excluded by carefully reading titles, abstracts, and full text. In addition, one study (Jiang et al., 2008) was excluded because another publication (Jiang and Tan, 2010) included their cases and controls. Therefore, 87 publications (Zintzaras, 2009; Aydin-Sayitoglu et al., 2006; Al-Achkar et al., 2014; Arruda et al., 2001; Allan et al., 2001; Abdalhabib et al., 2021; Alves et al., 2002; Al-Eitan et al., 2016; Bajpai et al., 2007; Balta et al., 2003; Bhat et al., 2012; Bhatla et al., 2008; Barnette et al., 2004; Bolufer et al., 2007; Baba et al., 2021; Bănescu et al., 2016; Bănescu et al., 2014; Chen et al., 1997; Chen et al., 2008; Crump et al., 2000; Chauhan et al., 2011; Chauhan et al., 2012; Canalle et al., 2004; Clavel et al., 2005; Chan et al., 2011; D'Alò et al., 2004; Davies et al., 2000; Davies et al., 2002; Dunna et al., 2013; Eyada et al., 2007; Feng et al., 2004; Farasani, 2019; Gra et al., 2008; Guven et al., 2015; Haase et al., 2002; Hishida et al., 2005; Haranatha Reddy and Jamil, 2006; Idris et al., 2020; Jiang and Tan, 2010; Joseph et al., 2004; Krajinovic et al., 1999; Lemos et al., 1999; Sasai et al., 1999; Naoe et al., 2000; Rollinson et al., 2000; Saadat and Saadat, 2000; Woo et al., 2000; Loffler et al., 2001; Wang et al., 2002; Yuille et al., 2002; Zhang et al., 2003; Seedhouse et al., 2004; Wang et al., 2004; Wu et al., 2004; Zou et al., 2004; Liu et al., 2005; Lourenco et al., 2005; Mondal et al., 2005; Pakakasama et al., 2005; Yang et al., 2005; Pigullo et al., 2007; Majumdar et al., 2008; Müller et al., 2008; Rimando et al., 2008; Souza et al., 2008; Suneetha et al., 2008; Taspinar et al., 2008; Ovsepian et al., 2010; Mandegary et al., 2011; Ouerhani et al., 2011; Suneetha et al., 2011; Kim et al., 2012; Li et al., 2012; Lordelo et al., 2012; Özten et al., 2012; Zhou et al., 2013; Zi et al., 2014; Kassogue et al., 2015; Lopes et al., 2015; Nasr et al., 2015; Weich et al., 2015; Kreile et al., 2016; Weich et al., 2016; Liu et al., 2017; Zehra et al., 2018; Muddathir et al., 2019; Rostami et al., 2019) were selected for the current study (Figure 1). Of these, there were 104 studies from 86 publications (14,100 leukemia cases and 23,793 controls, Table 1, Supplementary Table S1) for the *GSTM1* null genotype, 94 studies from 79 publications (12,928 leukemia cases and 22,036 controls, Table 2, Supplementary Table S1) for the *GSTT1* null genotype, and 33 studies from 30 publications (4,613 leukemia cases and 6,826 controls, Supplementary Table S1) for the combined effects of the *GSTM1* present/null and *GSTT1* present/null polymorphisms. In addition, there were 74 high-quality studies for the *GSTM1* null genotype, 71 high-quality studies for the *GSTT1* null genotype, and 25 high-quality studies for the combined effects of the *GSTM1* and *GSTT1* polymorphisms, as shown in Supplementary Table S1.

**TABLE 1:** The results of the association of the *GSTM1* polymorphism with the risk of leukemia.

Variable	*n*	Cases/controls	Test of association	Test of heterogeneity		FPRP	BFDP
			OR (95%CI)	*P*_h_	*I* ^2^ (%)		
Overall	104	14,100/23,793	**1.24 (1.14, 1.34)**	<0.001	65.0	**<0.001**	0.006
Type of leukemia							
ALL	39	4,744/7,653	**1.24 (1.09, 1.41)**	<0.001	58.9	0.508	0.977
AML	35	5,889/10,335	**1.26 (1.09, 1.45)**	<0.001	71.6	0.559	0.979
CML	20	2,141/3,713	1.20 (0.97, 1.48)	<0.001	68.7	NA	NA
Ethnicity							
Asian	25	3,267/6,133	**1.48 (1.30, 1.68)**	0.042	35.4	**<0.001**	**<0.001**
Caucasian	49	7,141/11,369	**1.14 (1.03, 1.26)**	<0.001	57.8	0.911	0.998
Indian	14	1,497/2,377	**1.37 (1.01, 1.87)**	<0.001	80.0	0.985	0.998
Mixed	14	2,126/3,510	0.99 (0.78, 1.26)	<0.001	72.3	NA	NA
Geographic region							
East Asia	22	2,915/5,576	**1.46 (1.27, 1.67)**	0.031	39.3	**<0.001**	**0.002**
Europe	24	3,888/7,347	1.06 (0.97, 1.14)	0.113	26.8	NA	NA
North Africa	6	482/578	**2.39 (1.28, 4.47)**	0.003	72.7	0.989	0.992
North America	9	2,109/2,630	1.10 (0.94, 1.30)	0.14	34.8	NA	NA
South America	9	924/2,225	0.92 (0.63, 1.35)	<0.001	78.3	NA	NA
South Asia	14	1,497/2,377	**1.37 (1.01, 1.87)**	<0.001	80.0	0.985	0.998
Southeast Asia	3	352/557	**1.68 (1.24, 2.27)**	0.592	0.0	0.760	0.949
West Asia	17	1,933/2,503	1.17 (0.93, 1.47)	<0.001	69.1	NA	NA

CML: chronic myeloid leukemia; AML: acute myeloid leukemia; ALL: acute lymphoblastic leukemia; NA: not available.

The bold values indicate significant results.

**TABLE 2:** The results of the association of the *GSTT1* polymorphism with the risk of leukemia.

Variable	*n*	Cases/controls	Test of association	Test of heterogeneity		FPRP	BFDP
			OR (95%CI)	*P*_h_	*I* ^2^ (%)		
Overall	94	12,928/22,036	**1.39 (1.23, 1.57)**	<0.001	78.2	**<0.001**	**0.008**
Type of leukemia							
ALL	36	4,586/7,143	**1.32 (1.12, 1.56)**	<0.001	64.3	0.546	0.974
AML	32	4,994/9,331	**1.38 (1.18, 1.62)**	<0.001	65.5	**0.062**	**0.778**
CML	19	2,130/3,687	1.53 (0.93, 2.51)	<0.001	92.1	NA	NA
Ethnicity							
Asian	23	3,172/5,956	**1.26 (1.15, 1.38)**	0.179	21.2	**0.001**	**0.052**
Caucasian	42	6,716/10,179	**1.37 (1.09, 1.72)**	<0.001	86.4	0.895	0.993
Indian	14	1,497/2,374	**1.78 (1.31, 2.42)**	<0.001	68.1	0.630	0.874
Mixed	13	1,474/3,123	1.28 (0.93, 1.75)	<0.001	69.4	NA	NA
Geographic region							
East Asia	20	2,820/5,479	**1.30 (1.15, 1.46)**	0.172	23.0	**0.009**	**0.367**
Europe	19	3,587/6,434	1.25 (0.87, 1.81)	<0.001	90.9	NA	NA
North Africa	6	482/578	**2.16 (1.01, 4.62)**	<0.001	80.0	0.996	0.998
North America	8	1,459/2,117	0.94 (0.73, 1.22)	0.155	34.2	NA	NA
South America	9	924/2,325	1.26 (0.91, 1.73)	0.021	55.6	NA	NA
South Asia	14	1,497/2,374	**1.78 (1.31, 2.42)**	<0.001	68.1	0.630	0.874
Southeast Asia	3	352/477	1.09 (0.79, 1.49)	0.327	10.6	NA	NA
West Asia	15	1,807/2,252	**1.57 (1.10, 2.22)**	<0.001	78.3	0.996	0.994

CML: chronic myeloid leukemia; AML: acute myeloid leukemia; ALL: acute lymphoblastic leukemia; NA: not available.

The bold values indicate significant results.

### Quantitative Synthesis

Overall, the *GSTM1* null genotype was associated with a significantly increased leukemia risk (OR = 1.24, 95% CI: 1.14–1.34, Table 1) when all the eligible studies were merged. Then, subgroup analysis was conducted by type of leukemia, and significantly increased acute lymphoblastic leukemia (ALL) (OR = 1.24, 95% CI: 1.09–1.41) and acute myeloid leukemia (AML) (OR = 1.26, 95% CI: 1.09–1.45) risk were also observed for the *GSTM1* null genotype. In addition, the *GSTM1* null genotype was associated with a significantly increased leukemia risk in Asians (OR = 1.48, 95% CI: 1.30–1.68), Caucasians (OR = 1.14, 95% CI: 1.03–1.26), and Indians (OR = 1.37, 95% CI: 1.01–1.87). Moreover, significantly increased leukemia risk was found for the *GSTM1* null genotype among countries of East Asia (OR = 1.46, 95% CI: 1.27–1.67), North Africa (OR = 2.39, 95% CI: 1.28–4.47), South Asia (OR = 1.37, 95% CI: 1.01–1.87), and Southeast Asia (OR = 1.68, 95% CI: 1.24–2.27), as shown in Table 1. Moreover, we found that the *GSTM1* null genotype frequencies were different in the different populations (Africans: 29.7%, Asians: 53.7%, Caucasians: 49.5%, and Indians: 35.8%) for the control groups.

Overall, the *GSTT1* null genotype was associated with a significantly increased leukemia risk (OR = 1.39, 95% CI: 1.23–1.57, Table 2). Then, subgroup analysis was conducted by type of leukemia, and significantly increased ALL (OR = 1.32, 95% CI: 1.12–1.56) and AML (OR = 1.38, 95% CI: 1.18–1.62) risk were observed for the *GSTT1* null genotype. In addition, the *GSTT1* null genotype was associated with significantly increased leukemia risk in Asians (OR = 1.26, 95% CI: 1.15–1.38), Caucasians (OR = 1.37, 95% CI: 1.09–1.72), and Indians (OR = 1.78, 95% CI: 1.31–2.42). Moreover, significantly increased leukemia risk was found for the *GSTT1* null genotype among countries of East Asia (OR = 1.30, 95% CI: 1.15–1.46), North Africa (OR = 2.16, 95% CI: 1.01–4.62), South Asia (OR = 1.78, 95% CI: 1.31–2.42), and West Asia (OR = 1.57, 95% CI: 1.10–2.22), as shown in Table 2. Moreover, we also observed that *GSTT1* null genotype frequencies were also different in the different races (Africans: 25.9%, Asians: 44.5%, Caucasians: 19.5%, and Indians: 15.6%) for the control groups.

**FIGURE 2:**
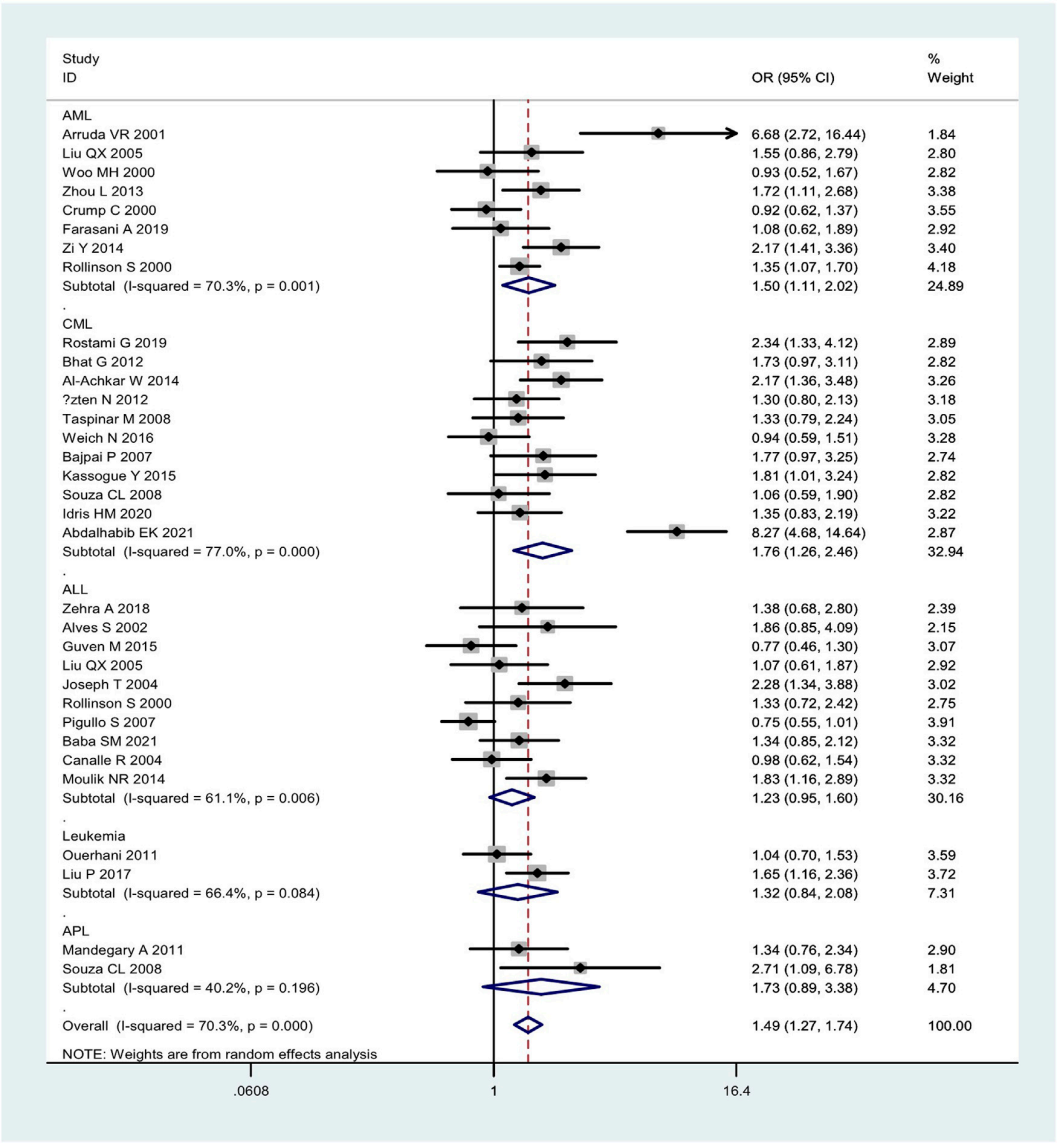
Forest plot of the combined effects of the *GSTMI* and *GSTTI* null genotypes with risk of leukemia in overall analysis and subgroup analysis by type of leukemia.

**FIGURE 3:**
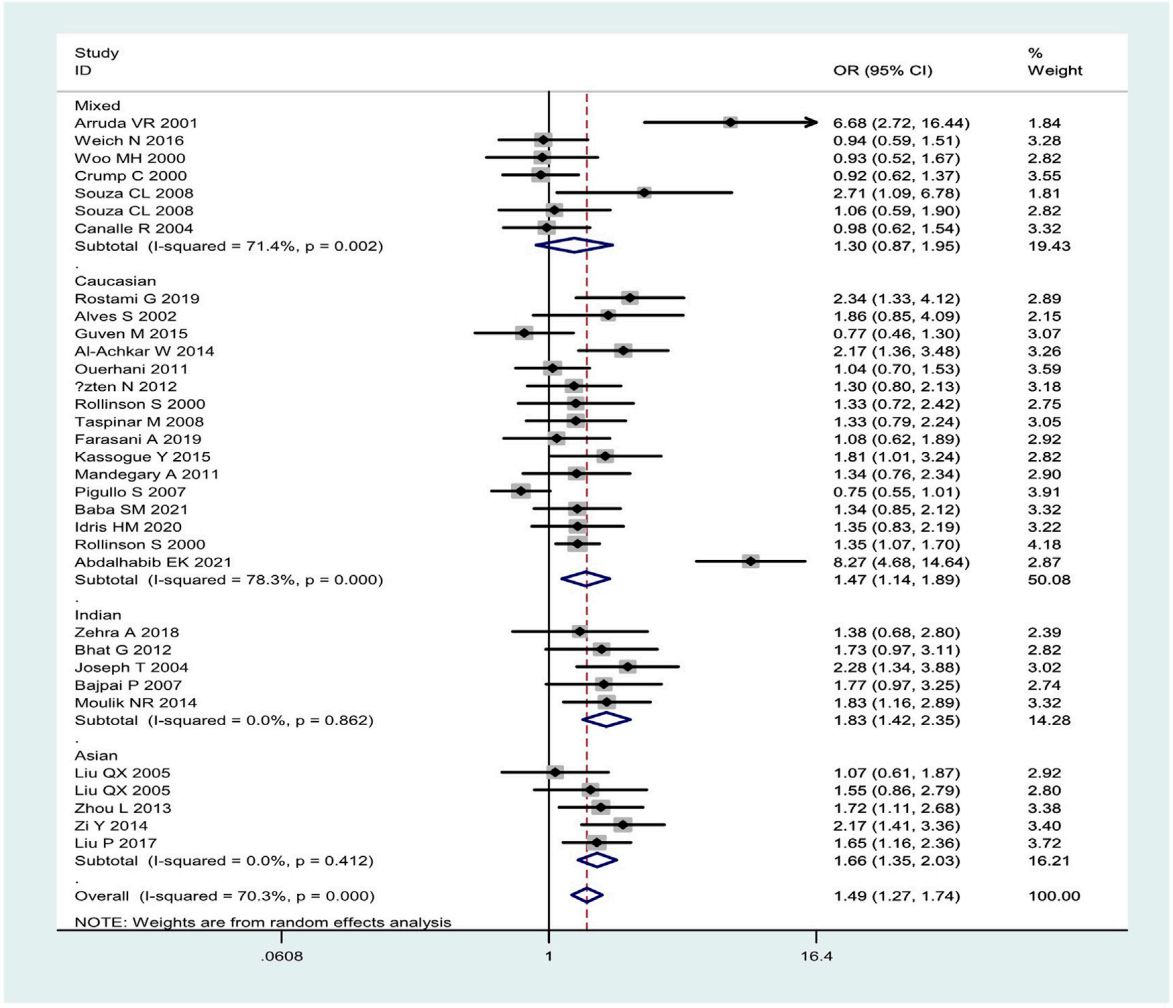
Forest plot of the combined effects of the *GSTM1* and *GSTT1* null genotypes with risk of leukemia in overall analysis and subgroup analysis by ethnicity.

**FIGURE 4:**
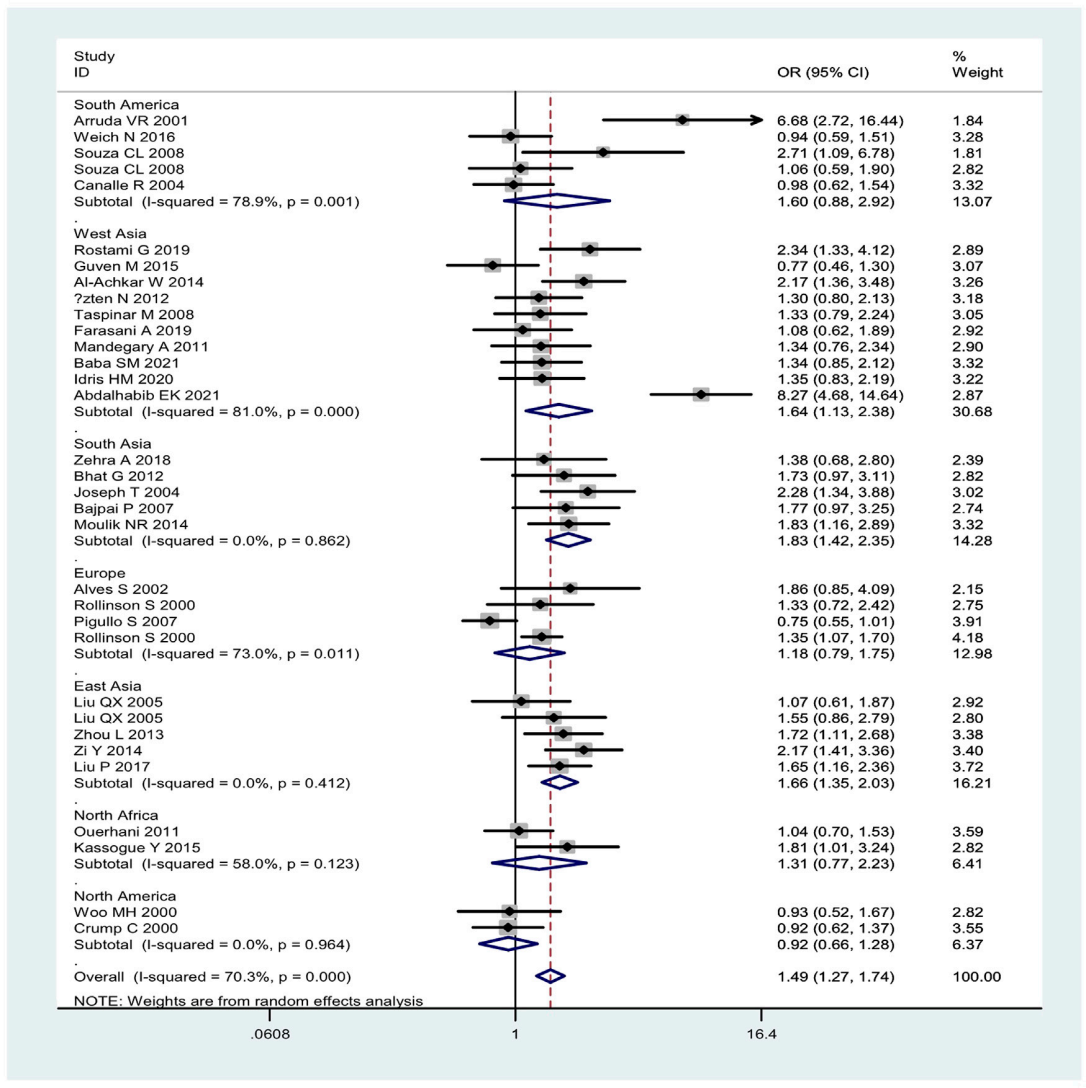
Forest plot of the combined effects of the *GSTM1* and *GSTT1* null genotypes with risk of leukemia in overall analysis and subgroup analysis by geographic region.

Overall, the combined effects of the *GSTM1* present/null and *GSTT1* present/null genotypes were associated with a significantly increased leukemia risk (OR = 1.49, 95% CI: 1.27–1.74, Figure 2). Then, subgroup analysis was conducted by type of leukemia, and significantly increased chronic myeloid leukemia (CLL) (OR = 1.76, 95% CI: 1.26–2.46) and AML (OR = 1.50, 95% CI: 1.11–2.02) risk was observed (Figure 2). In addition, the combined effects were associated with significantly increased leukemia risk in Asians (OR = 1.66, 95% CI: 1.35–2.03), Caucasians (OR = 1.47, 95% CI: 1.14–1.89), and Indians (OR = 1.83, 95% CI: 1.42–2.35), as shown in Figure 3. Moreover, significantly increased leukemia risk was found for the combined effects among countries of East Asia (OR = 1.66, 95% CI: 1.35–2.03), South Asia (OR = 1.83, 95% CI: 1.42–2.35), and West Asia (OR = 1.64, 95% CI: 1.13–2.38), as shown in Figure 4. Moreover, we also found that the risk genotypes frequencies of the combined effects of the *GSTM1* and *GSTT1* polymorphisms were also different in the different races (Asians: 46.8%, Caucasians: 54.0%, and Indians: 39.3%) for the control groups.

### Heterogeneity and Sensitivity Analyses

Between-studies heterogeneity was observed, as shown in Tables 1, 2 and Figures 2–4. A meta-regression analysis showed that the quality score of included studies (*p* = 0.007) were sources of heterogeneity for the *GSTM1* null genotype. For the *GSTT1* null genotype and combined effects, meta-regression analyses did not find sources of heterogeneity. Moreover, we did not observe any change when one study and low-quality studies were excluded from the overall analysis.

### Publication Bias

**FIGURE 5:**
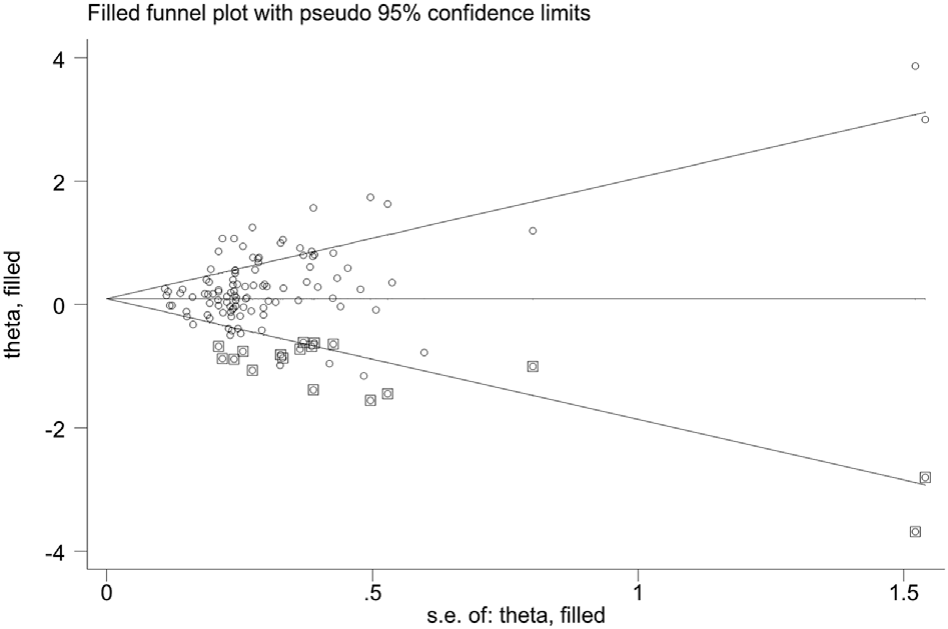
The Duval and Tweedie nonparametric “trim and fill” method’s funnel plot of the *GSTM1* present/null polymorphism with risk of leukemia.

**FIGURE 6:**
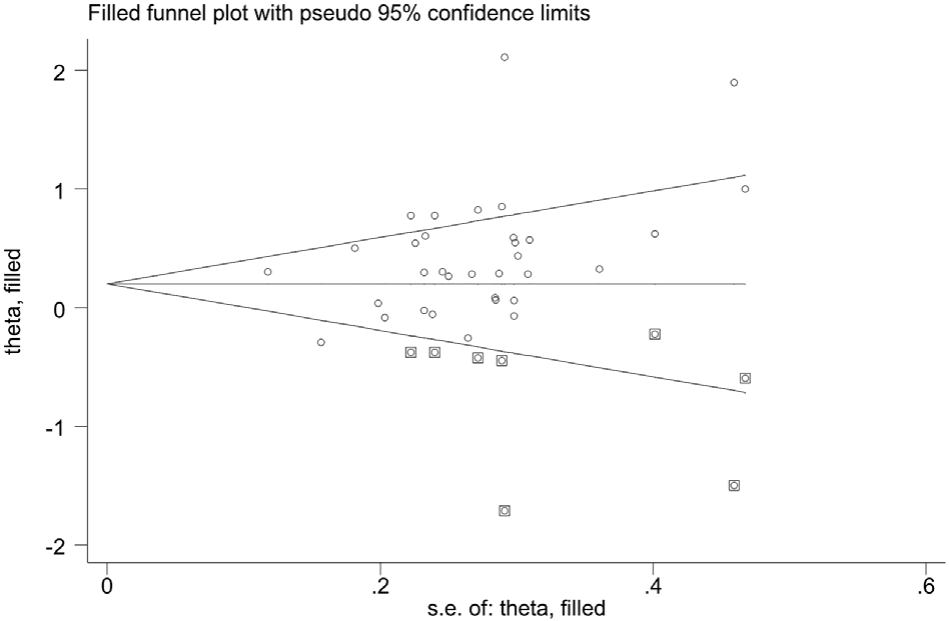
The Duval and Tweedie nonparametric “trim and fill” method’s funnel plot of the combined effects of the *GSTM1* present/null and *GSTTI* present/null polymorphisms with risk of leukemia.

Publication bias was found for *GSTM1* null genotype (*p* = 0.005) and the combined effects of *GSTM1* and *GSTT1* (*p* = 0.035), according to Begg’s funnel plot shape and Egger’s test in the current meta-analysis. Figures 5, 6 show the funnel plots of the nonparametric “trim and fill” method. We need to add 18 articles in the future for the *GSTM1* present/null polymorphism with risk of leukemia (Figure 5). Moreover, we need to add eight studies for the combined effects of the *GSTM1* present/null and *GSTT1* present/null polymorphisms on the risk of leukemia (Figure 6). However, the results did not change, indicating that the current study was stable in overall analysis when the nonparametric “trim and fill” method was applied.

### Test of Significant Associations in the Current Study

To investigate the false-positive results, FPRP and BFDP were applied. For the *GSTM1* null genotype, significant associations were considered as “positive” results in overall population (FPRP < 0.001 and BFDP = 0.006), Asians (FPRP < 0.001 and BFDP < 0.001), and East Asian population (FPRP < 0.001 and BFDP = 0.002), as shown in Table 1. For the *GSTT1* null genotype, significant associations were regarded as “positive” results in overall population (FPRP < 0.001 and BFDP = 0.008), AML (FPRP = 0.062 and BFDP = 0.778), Asians (FPRP = 0.001 and BFDP = 0.052), and East Asian population (FPRP = 0.009 and BFDP = 0.367), as shown in Table 2. For the combined effects of the *GSTM1* and *GSTT1* polymorphisms, significant associations were also considered as “positive” results in overall analysis (FPRP = 0.001 and BFDP = 0.027), Asians (FPRP = 0.005 and BFDP = 0.040), Indians (FPRP = 0.035 and BFDP = 0.095), and East Asian population (FPRP = 0.014 and BFDP = 0.040).

## Discussion

Overall, the individual *GSTM1* and *GSTT1* null genotypes and combined effects of the two genes were associated with significantly increased leukemia risk in the overall analysis and several subgroup analyses, such as Asians, Caucasians, and so on. However, the current study applied several subgroup analyses at the expense of multiple comparisons. Therefore, FPRP and BFDP values were applied to conduct the test of false-positive results.

Glutathione S-transferases (GSTs) are a group of enzymes that play vital roles in regulating the cellular detoxification of various exogenous carcinogens (Di Pietro et al., 2010). Moreover, it is believed that GSTs can protect cells against oxidative stress and its associated DNA damage (Singh, 2015). Furthermore, it is biologically plausible that subjects carrying these null genotypes may suffer a higher risk of developing multiple malignancies because their GST proteins do not function properly. Therefore, it is widely accepted that alterations in GSTs play roles in the process associated with the etiology of cancers. Based on biochemical properties described for the *GSTM1* present/null and *GSTT1* present/null polymorphisms, we expected that the individual and the combined effects of the two genes were associated with the risk of leukemia in any population. However, we only found that the individual *GSTM1* and *GSTT1* null genotypes and combined effects of the two genes are associated with increased leukemia risk in Asians, especially in the East Asian population, and the combined effects of the two genes are also associated with increased leukemia risk in Indians when we used the FPRP and BFDP values. These results showed that the same genes may play different roles in leukemia susceptibility in different races and countries because leukemia is a complicated multigenetic disease and different genetic backgrounds and environmental factors may contribute to the discrepancy (Begg and Mazumdar, 1994). Moreover, we only found that the *GSTT1* null genotype was associated with increased AML risk. The result showed that the same polymorphism also may play different roles in a different type of leukemia. Moreover, some results should be interpreted with caution, and it was necessary that a well-designed large sample study was conducted to explore the true association, such as in Southeast Asian and North African populations. Furthermore, publication bias was observed between the *GSTM1* null genotype and the combined effects of the two genes on the risk of leukemia. Figures 5, 6 showed that publication bias was caused according to low-quality small-sample studies. As far as we know, random error and bias were common for the small-sample–size studies, especially in molecular epidemiological studies. Moreover, small-sample studies were easier to publish if the results were significant as they tend to yield false-positive results because they may be not rigorous and were often of low quality.

Fourteen meta-analyses (Ye and Song, 2005; Das et al., 2009; Zintzaras, 2009; Vijayakrishnan and Houlston, 2010; Tang et al., 2013; He et al., 2014a; He et al., 2014b; Ma et al., 2014; Moulik et al., 2014; Tang et al., 2014; Xu and Cao, 2014; Li et al., 2018; Zhao et al., 2018; Wang et al., 2019) reported the individual effects of the *GSTM1* and *GSTT1* polymorphisms with leukemia risk. Wang et al. (2019) observed that the *GSTM1* and *GSTT1* null genotypes were significantly associated with elevated individual susceptibility to acute lymphoblastic leukemia (ALL) and AML; the *GSTT1* null genotype was also significantly associated with elevated individual susceptibility to chronic leukemia; the *GSTM1* and *GSTT1* null genotypes were significantly associated with elevated individual susceptibility to leukemia in Caucasians and West Asians; the *GSTM1* null genotype was also significantly correlated with elevated individual susceptibility to leukemia in East Asians. Li et al. (2018) found that the *GSTM1* and *GSTT1* polymorphisms were both significantly correlated with hematological malignancy in Caucasians, East Asians, and West Asians, and positive results were found for the *GSTM1* and *GSTT1* polymorphisms in patients with certain types of acute leukemia. Zhao et al. (2018) found that the *GSTM1* null genotype was associated with increased childhood ALL risk and the *GSTT1* null genotype was not associated with childhood ALL risk. He et al. (2014a) revealed that the *GSTM1* null genotype was associated with an increased risk of AML in East Asians and the *GSTT1* null genotype in Caucasians. Tang et al. (2014) suggested that the *GSTM1* and *GSTT1* null genotypes might be a potential risk factor for acute leukemia in Asians. He et al. (2014b) indicated that the *GSTT1* null genotype and the double-null *GSTT1* and *GSTM1* genotypes were associated with an increased risk of CML. Xu and Cao (2014) found that the *GSTT1* null variant was significantly associated with susceptibility to childhood ALL in Asians. Tang et al. (2013) found that the *GSTM1* null polymorphism was caused by childhood acute leukemia susceptibility. Ma et al. (2014) found that the *GSTM1* null genotype was significantly associated with increased risk of childhood acute leukemia in the Chinese population. Vijayakrishnan and Houlston (2010) found that the *GSTM1* null genotype was significantly associated with increased risk of childhood acute lymphoblastic leukemia, but should be interpreted with caution. Zintzaras (2009) suggested that no significant association was found between the *GSTM1* null genotype and CML risk, while the *GSTT1* null genotype was associated with increased risk of CML, especially in Indians. Das et al. (2009) indicated that significant increased risk of AML was observed with the *GSTM1* null genotype, while borderline significance was seen with the *GSTT1* null genotype. Ye and Song (2005) found that the *GSTM1* and *GSTT1* null genotypes appeared to be associated with a modest increase in the risk of ALL. Moulik et al. (2014) found that the *GSTM1* null genotype was associated with increased childhood ALL risk. These results might be not credible because many original studies were not included in previously published meta-analyses. Moreover, previously published meta-analyses did not conduct the false-positive test using FPRP and BFDP values. Therefore, we performed the current study to further explore these associations.

The present study had several limitations. First, only published studies were selected. Second, the confounding factors closely related to the outcome were not controlled, such as gender, smoking, and some other factors. The current study also has several advantages over previously published meta-analyses. First, the sample size was larger. Second, we investigate the false-positive results by applying the FPRP and BFDP values.

In summary, this study strongly indicates that the individual *GSTM1* and *GSTT1* null genotypes and combined effects of the two genes are associated with increased leukemia risk in Asians, especially in the East Asian population; the *GSTT1* null genotype is associated with increased AML risk; the combined effects of the two genes are associated with increased leukemia risk in Indians.

## Data Availability

The original contributions presented in the study are included in the article/Supplementary Material; further inquiries can be directed to the corresponding author.

## Abbreviations

BFDP, Bayesian false discovery probability; CIs, confidence intervals; CML, chronic myeloid leukemia; FPRP, false-positive report probabilities; GSTs, glutathione S-transferases; HWE, Hardy–Weinberg equilibrium; ORs, odds ratios.
