# Supplementary material for: Association Between the Individual and Combined Effects of the GSTM1 and GSTT1 Polymorphisms and Risk of Leukemia: A Meta-Analysis
Source: Front Genet. 2022 Jul 22;13:898937. doi: 10.3389/fgene.2022.898937 (PMC9355274; doi:10.3389/fgene.2022.898937)
Supplement: Supplementary file 1 [file Table2.docx]

**S2 Table** Scale for quality assessment of molecular association studies

| Criterion | Score |
| --- | --- |
| Source of case | |
| Selected from population or cancer registry | 2 |
| Selected from hospital | 1 |
| Not described | 0 |
| Source of control | |
| Population-based | 2 |
| Hospital-based | 1 |
| Not described | 0 |
| Ascertainment of leukemia | |
| Histological or pathological confirmation | 2 |
| Diagnosis by patient medical record | 1 |
| Not described | 0 |
| Ascertainment of control | |
| Controls were tested to screen out leukemia | 2 |
| Controls were subjects who did not report leukemia, no objective testing | 1 |
| Not described | 0 |
| Matching | |
| Controls matched with cases by age and sex | 2 |
| Controls matched with cases only by age or sex | 1 |
| Not matched or not described | 0 |
| Genotyping examination | |
| Genotyping done blindly and quality control | 2 |
| Only genotyping done blindly or quality control | 1 |
| Unblinded and without quality control | 0 |
| Association assessment | |
| Assess association between genotypes and lung cancer with appropriate statistics and adjustment for confounders | 2 |
| Assess association between genotypes and lung cancer with appropriate statistics without adjustment for confounders | 1 |
| Inappropriate statistics used | 0 |
| Total sample size |  |
| >1000 | 2 |
| 200-1000 | 1 |
| <200 | 0 |

HWE: Hardy-Weinberg equilibrium
